# Genetic diversity and delineation of *Salmonella* Agona outbreak strains by next generation sequencing, Bavaria, Germany, 1993 to 2018

**DOI:** 10.2807/1560-7917.ES.2019.24.18.1800303

**Published:** 2019-05-02

**Authors:** Alexandra Dangel, Anja Berger, Ute Messelhäußer, Regina Konrad, Stefan Hörmansdorfer, Nikolaus Ackermann, Andreas Sing

**Affiliations:** 1 Bavarian Health and Food Safety Authority (LGL), Oberschleissheim, Germany; 2 These authors contributed equally to this article

**Keywords:** Salmonella, food-borne infections, molecular methods, surveillance, outbreaks, public health, Europe, Germany, food-borne infections, bacterial infections, salmonellosis, surveillance

## Abstract

**Background:**

In 2017, a food-borne *Salmonella* Agona outbreak caused by infant milk products from a French supplier occurred in Europe. Simultaneously, *S.* Agona was detected in animal feed samples in Bavaria.

**Aim:**

Using next generation sequencing (NGS) and three data analysis methods, this study’s objectives were to verify clonality of the Bavarian feed strains, rule out their connection to the outbreak, explore the genetic diversity of Bavarian *S.* Agona isolates from 1993 to 2018 and compare the analysis approaches employed, for practicality and ability to delineate outbreaks caused by the genetically monomorphic Agona serovar.

**Methods:**

In this observational retrospective study, three 2017 Bavarian feed isolates were compared to a French outbreak isolate and 48 *S.* Agona isolates from our strain collections. The later included human, food, feed, veterinary and environmental isolates, of which 28 were epidemiologically outbreak related. All isolates were subjected to NGS and analysed by: (i) a publicly available species-specific core genome multilocus sequence typing (cgMLST) scheme, (ii) single nucleotide polymorphism phylogeny and (iii) an in-house serovar-specific cgMLST scheme. Using additional international *S.* Agona outbreak NGS data, the cluster resolution capacity of the two cgMLST schemes was assessed.

**Results:**

We could prove clonality of the feed isolates and exclude their relation to the French outbreak. All approaches confirmed former Bavarian epidemiological clusters.

**Conclusion:**

Even for *S.* Agona, species-level cgMLST can produce reasonable resolution, being standardisable by public health laboratories. For single samples or homogeneous sample sets, higher resolution by serovar-specific cgMLST or SNP genotyping can facilitate outbreak investigations.

## Introduction

Salmonellosis is one of the most common food-borne human diseases. It is often transmitted via contaminated meat, eggs or seafood products. Moreover, due to the robustness of *Salmonella* spp., dried products like herbs or spices have also proven their potential as vehicle of infection. Of more than 2,600 different serovars of *S. enterica*, only a few non-typhoidal serovars are responsible for most human infections. In *S. enterica *subsp.* enterica*, these serovars include for example Enteridis, Typhimurium and Agona. In Europe, *S.* Agona is far from leading the list of pathogenic serovars, as cases of *S. *Enteritidis and *S. *Typhimurium are much more numerous [1]. Globally, *S*. Agona is a common pathogen and food-borne outbreaks connected to it have been consistently reported in several countries. Examples are a 2002−03 outbreak, caused by aniseed-fennel-caraway tea products affecting 77 patients in Germany [2,3], a 2008 outbreak connected to meat products of one supplier causing 163 infections in 10 different countries with most cases in the United Kingdom [4], a 2011 multi-state outbreak in the United States (US) caused by fresh papaya resulting in more than 100 infected patients [5], or a point-sourced outbreak caused by tuna sushi in Sydney, Australia in 2015 [6].

In outbreak investigations, serotyping and phage typing have been used for decades in many laboratories including reference laboratories. Serotyping is still serving as a gold-standard technique for routine typing. In combination with other typing techniques like phage typing, it may be suited for the investigation of small, geographically limited outbreaks [7]. However, many serovars are polyphyletic and serotyping sometimes confounds genetically unrelated isolates and thus does not recognise evolutionary groupings in some cases. Therefore, attempts were made some years ago to replace this technique by molecular typing methods such as multilocus sequence typing (MLST), which is able to recognise evolutionary relationships with higher resolution [8]. Furthermore, since many years, pulsed-field gel electrophoresis (PFGE), classifying bacteria based on their universal band pattern after chromosomal restriction, is globally used as a standard molecular technique in outbreak investigations [9-11]. However, despite advantages of molecular techniques, traditional serotyping is still universally used and provides an important historical context.

Beyond PFGE and MLST, variable number of tandem repeats (VNTRs) proved to be suitable molecular targets for assessing genetic polymorphisms within bacterial species [12,13]. The multilocus variable number of tandem repeats (MLVA) technique, as a form of VNTR typing showed increased analysis depth in outbreak investigations and proved to be suitable for important *Salmonella* serovars such as Enteritidis, Typhimurium or Dublin [14-16]. However, the variability of protocols and targets hindered comprehensive standardisation, although efforts towards this are ongoing [17,18]. All these techniques provide reliable first level classification and are discriminative enough to investigate epidemiologically well-defined outbreaks.

Nonetheless, in the meantime, a number of studies have shown that whole genome sequencing (WGS) gives the highest resolution for outbreak investigation, especially if case distribution is diffuse with respect to geographical area or time frame of occurrence [7,19,20]. For implementation of WGS in *S.* Agona outbreak investigations and molecular surveillance, it has to be taken into account that this serovar is monophyletic, more homogeneous, as well as evolutionarily younger than most other well-investigated pathogenic serovars [21,22].

In 2017, three feed samples (rapeseed meal) of a factory in the district of Lower Bavaria were submitted to the Bavarian Health and Food Safety Authority. Culture-based species and serovar identification detected *S.* Agona in all three samples. In December 2017, an outbreak of the same serovar was reported in France, attributable to 37 French cases and two international cases, caused by infant milk products of a French supplier and traceable to one single French production facility [23,24].

The aim of the current WGS investigation, using next generation sequencing (NGS) was to verify potential clonality of the Bavarian isolates, to exclude any connection between the Bavarian feed samples and the simultaneous French outbreak and to gain a more precise insight into the genetic diversity of *S.* Agona collected in Bavaria over the past 25 years.

## Methods

### Isolates and sequence data used in the analyses

For this observational retrospective study, we used 48 *S*. Agona isolates dated from 1993 to 2018 from our strain collections to investigate the three isolates of the Bavarian rapeseed meal from 2017 in a wider context. The total 51 isolates were all the *S*. Agona isolates available for us. As many diagnostic laboratories exist in Bavaria, our isolates were not representative for the occurrence of *S.* Agona in this federal state, where, for example, 99 cases of human infections had been officially notified in the 2011 to 2018 period alone. 

The 48 isolates that we employed were either from humans, food, feed, animals or the environment. A total of 28 thereof were outbreak-related. Outbreak isolates belonged to four epidemiologically-linked events represented by 15, three, three and two isolates as well as an additional five isolates with a suspected epidemiological connection. 

The 48 isolates from 1993 to 2018 were studied by WGS together with the three 2017 isolates of the Bavarian rapeseed feed. Raw NGS data of the published representative isolate of the French outbreak [24], available under European Nucleotide Archive (ENA) accession ERR2219379, were also added to the bioinformatics analyses. 

For the evaluation of necessary analysis depth, 70 NGS raw datasets from various European outbreaks, published by Zhou et al. 2013 [21], available under National Center for Biotechnology Information (NCBI) bioproject PRJEB1944, were added to the data analysis as well. 

To distinguish the subset of isolates derived from our strain collections, or data thereof, from the data published by Zhou et al. [21], we further refer to our 51 *S.* Agona isolates/strains as ‘Bavarian’.

### Species and serovar identification

All Bavarian *S.* Agona strains were cultured on Columbia sheep blood agar (Oxoid, Wesel, Germany) and identified by matrix-assisted laser desorption/ionisation time-of-flight mass spectroscopy (MALDI-TOF MS; Bruker, Bremen, Germany). Somatic (O) and flagellar (H) antigens were identified by using slide agglutination (antisera provided by Sifin, Berlin, Germany) according to the White−Kauffmann−Le Minor scheme [25].

### Next generation sequencing 

*Salmonella* Agona isolates were freshly grown on blood agar plates. One inoculation loop of bacterial material was suspended in 50 µL phosphate buffered saline (PBS) and cells were pre-treated with 1 µg lysozyme for 15 min at 37 °C followed by a 2 hour incubation step at 65 °C with 200 µL incorporation buffer, 200 µL lysis buffer, 30 µL 20 mg/mL Proteinase K and 10 µL 10 mg/mL ribonuclease (RNase) A (all reagents from Promega, Mannheim, Germany). Genomic DNA (gDNA) was then isolated with the Maxwell 16 LEV Blood DNA Kit on the Maxwell 16 instrument (Promega, Mannheim, Germany) according to manufacturer’s instructions with Tris buffer for gDNA elution.

Whole genome libraries for NGS were prepared using the Nextera XT kit (Illumina, San Diego, California, US). Next generation sequencing was performed on the Illumina MiSeq with 2x250 bp paired-end reads. Sequencing runs were evaluated for quality using the Illumina SAV Software.

Sequencing data were uploaded to the NCBI sequence read archive (SRA) [26], under BioProject PRJNA473689.

### Multilocus sequence typing analyses

Core genome MLST (cgMLST) of reads was performed with Ridom SeqSphere + Software (Ridom, Munster, Germany [27]) with default settings for trimming and velvet assembly. For the assignment of cgMLST alleles, two different schemes were used: (i) a publicly-available *S. enterica* (species level) cgMLST scheme designed by Enterobase and (ii) an in-house serovar-specific cgMLST scheme. 

#### Enterobase-designed *Salmonella enterica* core genome multilocus typing scheme

The publicly available species-specific SeqSphere + software-implemented *S. enterica* cgMLST scheme with the 3,002 target loci developed as *Salmonella* cgMLST v2 scheme by Enterobase was employed to analyse sequencing data [28,29]. 

#### In-house serovar-specific *Salmonella* Agona core genome multilocus typing scheme

An in-house developed serovar-specific *S.* Agona scheme, based on reference genome NC_011149.1 of strain SL-483 and query genomes NC_022991.1, NZ_CP015024.1, NZ_CP011259.1, was generated using the following default filter thresholds. 

For the reference genome filter thresholds: (i) minimum length: 60 bases; (ii) start codon and single stop codon required at beginning and end of gene; (iii) homologous/paralogous gene filter, excluding multiple copies of a gene with basic local alignment search tool (BLAST) overlap ≥ 100 bp or identity ≥ 90%; (iv) overlap filter, excluding overlap with other genes > 4 bases. 

For the query genome filters thresholds: (i) start and stop codon required at beginning and end of gene; (ii) BLAST hit locus overlap = 100% and identity ≥ 90% in every query genome; (iii) BLAST options: word size = 11, mismatch penalty = −1, match reward = 1, gap open costs = 5, gap extension costs = 2.

Thereby, the final scheme resulted in 4,111 target loci. 

New alleles and sequence types (ST) were submitted to the nomenclature server for the public scheme.

#### Assessment of relationships between isolates’ core genome multilocus sequence type allelic profiles by minimum spanning trees

cgMLST typing results were visualised in minimum spanning trees (MSTs), excluding all samples in the respective scheme, not fulfilling ‘good target’ quality control (QC) for > 90% for the scheme’s target loci (= 90% of targets present in the isolate, same length as reference +/− 3 triplets, without ambiguities and without frame shifts in consensus, Table 1). A cluster was defined as a group of closely related cgMLST-analysed isolates in both schemes with a single-linkage threshold of ≤ 7 alleles. This was the default distance threshold for the software-implemented public *S.* *enterica* scheme and was adopted for direct comparison for the *S.* Agona scheme. During typing with the *S. enterica* cgMLST scheme, the SeqSphere + software assigns an existing cluster type (CT) to each isolate with an allelic distance ≤ 7 to an already established CT founder profile on the central nomenclature server. Otherwise, a new CT is established, uploaded and the isolate becomes the founder of this CT [30,31]. 

**Table 1:** Characteristics of sequenced Bavarian *Salmonella* Agona isolates and French representative outbreak isolate, Germany, 1993−2018 (n = 52)

Sample name	Year	Material	Isolation source	Country/state	Country of origin/travel history	*S*. *enterica* cgMLST CT	cgMLST cluster	Epidemiological link
ERR2219379^a^	2017	NA	Human	France	NA	704	ND	NA
SA0001	2011	Stool	Human	Germany/Bavaria	Iraq	1195	2	Travel cluster
SA0002	2011	Stool	Human	Germany/Bavaria	Iraq	1215	ND	NA
SA0004	2012	Stool	Human	Germany/Bavaria	Afghanistan	1195	2	Travel cluster
SA0005	2012	Stool	Human	Germany/Bavaria	Madagascar	1209	None	NA
SA0006	2013	Stool	Human	Germany/Bavaria	Germany	1210	ND	NA
SA0007	2013	Stool	Human	Germany/Bavaria	Russia	1211	ND	NA
SA0008	2013	Bacterial strain	Human	Germany/Bavaria	Germany	1212	ND	NA
SA0009	2013	Bacterial strain	Human	Germany/Bavaria	Germany	1199	6	NA
SA0011	2014	Stool	Human	Germany/Bavaria	Unspecified foreign country	1214	8	NA
SA0012	2014	Stool	Human	Germany/Bavaria	Syria	1195	2	Travel cluster
SA0013	2014	Stool	Human	Germany/Bavaria	Syria	1195	2	Travel cluster
SA0015	2014	Stool	Human	Germany/Bavaria	Syria	1194	ND	NA
SA0016	2014	Stool	Human	Germany/Bavaria	Nigeria	1195	2	Travel cluster
SA0017	2015	Stool	Human	Germany/Bavaria	Kosovo under UN Security Council Resolution 1244	1196	ND	NA
SA0018^b^	2015	Stool	Human	Germany/Bavaria	Eritrea	1214	8^b^	NA
SA0019	2015	Bacterial strain	Human	Germany/Bavaria	Colombia	1197	ND	NA
SA0031^b^	2018	Nutritional supplement	Nutritional supplement	Germany/Bavaria	NA	1513	ND^b^	NA
SA0032	2017	Stool	Human	Germany/Bavaria	NA	1198	ND	NA
SA0033	2017	Stool	Human	Germany/Bavaria	NA	1124	ND	NA
SA0034	2017	Stool	Human	Germany/Bavaria	NA	1199	6	NA
SA0035	2017	Cattle	Cattle	Germany/Bavaria	NA	1200	ND	NA
SA0036	2015	Chicken faeces	Environment	Germany/Bavaria	NA	1134	4	Chicken cluster
SA0037^b,c^	2015	Cattle	Cattle	Germany/Bavaria	NA	ND^c^	ND^b,c^	NA
SA0038	2015	Chicken faeces	Environment	Germany/Bavaria	NA	1134	4	Chicken cluster
SA0039	2015	Chicken faeces	Environment	Germany/Bavaria	NA	1134	4	Chicken cluster
SA0040	2015	Chicken	Environment	Germany/Bavaria	NA	1201	ND	NA
SA0041	2008	Black pepper	Spices	Germany/Bavaria	NA	1202	ND	NA
SA0042	2017	Animal feed	Animal feed	Germany/Bavaria	NA	1193	3	Feed cluster
SA0043	2017	Animal feed	Animal feed	Germany/Bavaria	NA	1193	3	Feed cluster
SA0044	2017	Animal feed	Animal feed	Germany/Bavaria	NA	1193	3	Feed cluster
SA0045	2003	Digestive tea	Tea/raw tea	Germany/Bavaria	NA	1203	1	Tea outbreak
SA0046	2003	Digestive tea	Tea/raw tea	Germany/Bavaria	NA	1203	1	Tea outbreak
SA0047	2003	Cough and bronchial tea	Tea/raw tea	Germany/Bavaria	NA	1203	1	Tea outbreak
SA0048	1994	Turkey leg	Food	Germany/Bavaria	NA	1204	ND	NA
SA0050	1994	Shredded coconut	Food	Germany/Bavaria	NA	1205	5	Coconut cluster
SA0051	1994	Shredded coconut	Food	Germany/Bavaria	NA	1207	7	NA
SA0052	1994	Shredded coconut	Food	Germany/Bavaria	NA	1205	5	Coconut cluster
SA0053	2003	Aniseed	Tea/raw tea	Germany/Bavaria	NA	1203	1	Tea outbreak
SA0054	2003	Children calming tea	Tea/raw tea	Germany/Bavaria	NA	1203	1	Tea outbreak
SA0055	2003	Children calming tea	Tea/raw tea	Germany/Bavaria	NA	1203	1	Tea outbreak
SA0056	2003	Aniseed	Tea/raw tea	Germany/Bavaria	NA	1203	1	Tea outbreak
SA0057^b^	2003	Aniseed organic	Tea/raw tea	Germany/Bavaria	NA	1203	1^b^	Tea outbreak
SA0058	2003	Aniseed organic	Tea/raw tea	Germany/Bavaria	NA	1203	1	Tea outbreak
SA0059	2003	Pectoral tea	Tea/raw tea	Germany/Bavaria	NA	1203	1	Tea outbreak
SA0060	2003	Flatulence tea	Tea/raw tea	Germany/Bavaria	NA	1203	1	Tea outbreak
SA0061	2003	Pectoral and cough tea	Tea/raw tea	Germany/Bavaria	NA	1203	1	Tea outbreak
SA0062	2003	Pectoral and cough tea	Tea/raw tea	Germany/Bavaria	NA	1203	1	Tea outbreak
SA0063	2003	Aniseed	Tea/raw tea	Germany/Bavaria	NA	1203	1	Tea outbreak
SA0064	2003	Aniseed	Tea/raw tea	Germany/Bavaria	NA	1203	1	Tea outbreak
SA0065	1993	Turkey	Food	Germany/Bavaria	NA	1207	7	NA
SA0066	2018	Stool	Human	Germany/Bavaria	Thailand, Cambodia, Vietnam	1206	ND	NA

cgMLST: core genome multilocus sequence typing; CT: cluster type; NA: no information available; ND: none detected; UN: United Nations.

^a^ Representative dataset of the French outbreak.

^b ^Excluded from cgMLST with *S.* Agona scheme.

^c^ Excluded from cgMLST with *S.* *enterica* scheme.

#### Multilocus sequence typing

In silico MLST analysis of NGS data was performed with the standard seven-gene target scheme [32].

### Single nucleotide polymorphism phylogeny

Whole genome (wg) single nucleotide polymorphism (SNP)-based phylogeny was calculated using the run_snp_pipeline script of the PHEnix pipeline by Public Health England [33]. It includes trimming with trimmomatic [34], mapping to the reference genome NC_011149.1 of strain SL-483 with bwa-mem mapping [35] with default settings, variant calling and filtering (frequency ≥ 0.9, mapping quality score ≥ 30, read depth ≥ 10) by Genome Analysis Toolkit (GATK)2 Unified Genotyper [36]. Variant calls for all SNP positions passing filters and all positions not passing filters were extracted and SNPs were concatenated to alignments with the vcf2fasta-script from the same pipeline, allowing ≤ 90% missing data per sample and ≤ 20% missing data per position throughout all samples. Maximum likelihood (ML) trees were generated from SNP alignments by RaxML [37], including 100 bootstrap replicates.

## Results

Next generation sequencing of the 51 Bavarian *S.* Agona isolates (named with prefix SA), collected between 1993 and 2018 revealed high quality reads and reference genome coverage of 28−171 fold. In our bioinformatics data analyses, we included the published NGS raw data (ENA accession: ERR2219379) of an isolate from a case of the infant-milk-caused outbreak originating from a France-based manufacturer [24]. This isolate served as a representative of the French outbreak. All isolates were typed by in silico MLST, resulting in ST 13, the typical ST for serovar Agona [8].

**Figure 1:**
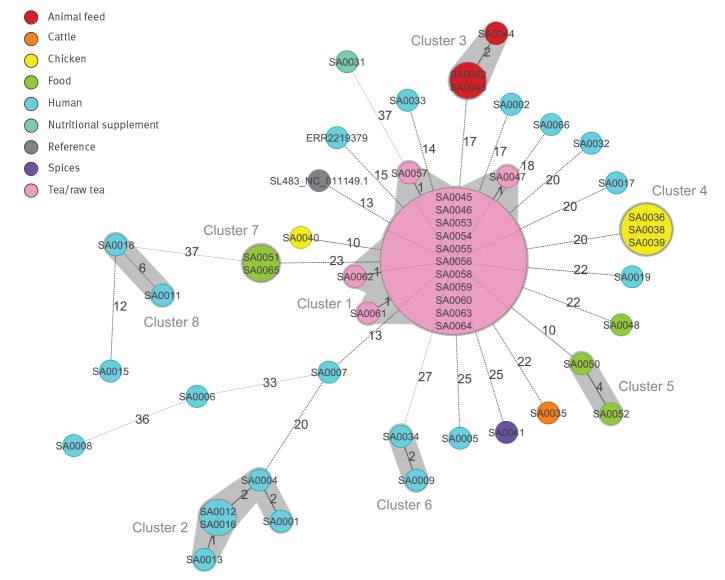
Minimum spanning tree of the core genome multilocus sequence type allelic profiles of *Salmonella *Agona strains, including 51 Bavarian isolates, a representative isolate of an infant-milk-caused outbreak in France and the reference strain SL-483, Germany, 1993−2018 (n = 53 isolates)

**Figure 2:**
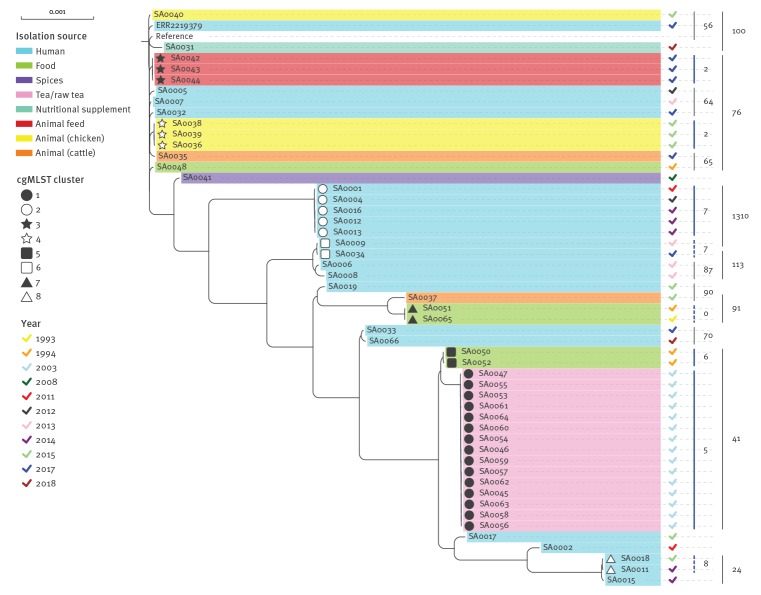
Maximum likelihood tree resulting from the whole genome single nucleotide polymorphism-based phylogenetic analysis of *Salmonella* Agona strains including 51 Bavarian isolates, a representative isolate of an infant milk-caused outbreak in France and the reference strain SL-483, Germany, 1993−2018 (n = 53 isolates)

In a next step, the isolates were typed with Ridom SeqSphere + software with the public *S.* *enterica* cgMLST scheme, consisting of Enterobase-developed 3,002 target loci (Figure 1). Additionally, wg SNP analysis was performed to investigate the phylogenetic relationship in highest possible resolution (Figure 2).

cgMLST: core genome multilocus sequence typing.

Bavarian *S. *Agona samples have the prefix SA. The representative isolate of the infant-milk outbreak in France [23] is ERR2219379. The National Center for Biotechnology Information (NCBI) reference genome of strain SL-483 has GenBank accession number NC_011149.1.

The analysis and tree were obtained with the public Ridom-SeqSphere + -integrated *S. enterica* cgMLST scheme of 3,002 target loci.

On the tree, allele distances between samples are indicated. Clusters of samples with maximum seven alleles distance are shaded in grey. Samples are colour coded by their isolation source, as given in the legend.

cgMLST: core genome multilocus sequence typing.

Bavarian *S. *Agona samples have the prefix SA. The representative isolate of the infant milk outbreak in France [23] is ERR2219379. The National Center for Biotechnology Information (NCBI) reference genome of strain SL-483 has GenBank accession number NC_011149.1 and figures in the tree as ‘reference’.

In the tree, samples are colour coded according to isolation source, *S.* Agona cgMLST cluster and collection year as given in the legend.

The general scale bar indicates 0.001 substitutions per site (597 SNPs), based on an alignment of 596,849 positions.

SNP distance bars between specific samples are indicated on the right side. Blue vertical SNP scale bars indicate epidemiologically-linked cgMLST clusters, blue vertical dashed lines indicate cgMLST clusters without epidemiological link and black vertical lines indicate exemplary distances between non clustered samples.

The MST from results of the *S. enterica* cgMLST scheme, including all samples exceeding the target-QC cut-off, revealed in total eight clusters with maximum six alleles difference (Figure 1). The four most relevant clusters (1−4) comprised three to 15 samples with a maximum within cluster difference of zero to five alleles (Table 2). Each of the remaining four clusters (5−8) included two samples and internal distances ranging from zero (cluster 7) to six alleles (cluster 8).

**Table 2:** Allele and single nucleotide polymorphism differences within and between clusters of the sequenced Bavarian *Salmonella *Agona isolates, Germany, 1993−2018 (n = 34 isolates)

Measure	Method	Cluster ID (number of isolates) Epidemiological link							
		Cluster 1 (15) Tea outbreak	Cluster 2 (5) Travel cluster	Cluster 3 (3) Feed cluster	Cluster 4 (3) Chicken cluster	Cluster 5 (2) Coconut cluster	Cluster 6 (2) None detected	Cluster 7 (2) None detected	Cluster 8^a^ (2)^a^ None detected
Within cluster distance: median (min–max)	*Salmonella enterica* cgMLST	0 (0–3)	2 (0–5)	0 (0–2)	0 (0–0)	4 (4–4)	2 (2–2)	0 (0–0)	6 (6–6)
	*S.* Agona cgMLST	0 (0–3)	2 (0–6)	1 (0–3)	0 (0–2)	3 (3–3)	2.5 (0–5)	0 (0–0)	None^a^
	SNP phylogeny	1 (0–5)	3.5 (0–7)	2 (0–2)	2 (0–2)	6 (6–6)	7 (7–7)	0 (0–0)	8 (8–8)
Min distance to nearest neighbour	*S. enterica* cgMLST	10	20	17	20	10	27	23	12
	*S.* Agona cgMLST	15	30	25	28	15	33	37	None^a^
	SNP phylogeny	21	47	37	37	21	49	59	22
Nearest neighbour outside cluster in question	*S. enterica* cgMLST	SA0040, SA0050 (in cluster 5)	SA0007	SA0053, SA0054, SA0055, SA0058 (all cluster 1)	SA0055 (in cluster 1)	SA0053, SA0054, SA0055, SA0058 (all cluster 1)	SA0053 (in cluster1)	SA0055, SA0056 (all cluster 1)	SA0015
	*S.* Agona cgMLST	SA0007, SA0050 (in cluster 5)	SA0047 (in cluster 1)	SA0047 and SA0058 (both in cluster 1), SA0007	SA0007	SA0055, SA0056 (all cluster 1)	SA0053 (in cluster 1)	SA0007	None^a^
	SNP phylogeny	SA0050 (in cluster 5)	SA0047 (in cluster 1)	SA0007	SA0007	SA0057 (in cluster 1)	SA0047 (in cluster 1)	SA0018^a^ (in cluster 8)	SA0015

cgMLST: core genome multilocus sequence typing; max: maximum; min: minimum; SNP: single nucleotide polymorphism.

^a^ Cluster 8 not detected in *S.* Agona cgMLST due to exclusion of sample SA0018.

None of the 51 Bavarian isolates collected from 1993–2018 clustered with the representative sample from the recent outbreak in France. The French representative sample ERR2219379 differed from the Bavarian samples in at least 15 alleles/40 SNPs. This difference was observed to distinguish unrelated isolates and clusters from different years or with different epidemiological origins throughout the whole sample set (Figure 1, Figure 2, Table 1).

The three Bavarian feed isolates collected in 2017 from rapeseed cake (SA0042, SA0043, SA0044) built up a distinct cluster with two alleles maximum distance (Figure 1, cluster 3), corresponding to two SNPs in the wg SNP analysis (Figure 2). They differed from the nearest neighbours outside the clusters in at least 17 alleles/37 SNPs (Table 2).

Furthermore, some of the Bavarian strains, isolated in former years, aggregated in epidemiologically described clusters, although the corresponding isolates available in our strain collection for sequencing mainly covered food or veterinary samples and corresponding human isolates were not available for analysis. Most eye-catching, the isolates of the biggest cluster with 15 samples (Figure 1, cluster 1) were all isolated from tea or raw tea products, connected to a diffuse outbreak caused by aniseed-fennel-caraway tea products in 2002–03. The outbreak investigation at that time identified contaminated raw tea imported from Turkey as source [2,3]. All isolates of this cluster were closely connected with zero to one allele single-linkage distance (Figure 1) and three alleles or five SNPs (Figure 2) maximum distance within the cluster. They showed at least 10 alleles/26 SNPs to all other non-connected isolates (Table 2).

The five isolates of cluster 2 with single-linkage distances of zero to two alleles and maximum intra-cluster distance of five alleles or seven SNPs were obtained from asylum seekers between 2011 and 2015 (Table 1). Details of their travel history regarding countries or period are unknown. Hence the slightly higher variation of their allelic/SNP distances than in other point-sourced epidemiologically linked clusters is not surprising. Cluster 4 consists of three samples with zero alleles/SNPs difference to each other but at least 20 alleles/45 SNPs difference to the other samples. Isolates in this cluster shared a clear epidemiological link, as samples originated from chicken faecal samples, collected in 2015 in different laying hen flocks of one Bavarian egg producer.

Clusters 5, 6, 7 and 8 only consist of two isolates each, respectively. Cluster 5 was built up from two shredded coconut samples from 1994 with four alleles/six SNPs difference which likely have an epidemiological link although information on their origin or supplier is not available. Isolates from two human patients without epidemiological link clustered together with two alleles (cluster 6) and seven SNPs distance. Cluster 7 came from two genetically identical strains from food samples from 1993−94 (shredded coconut and turkey) for which no epidemiological link is known. One additional pair of closely related human samples (SA0011 and SA0018) with reported travel history to Eritrea (SA0018) and an unspecified country (SA0011) built cluster 8 with six alleles and eight SNPs difference.

**Figure 3:**
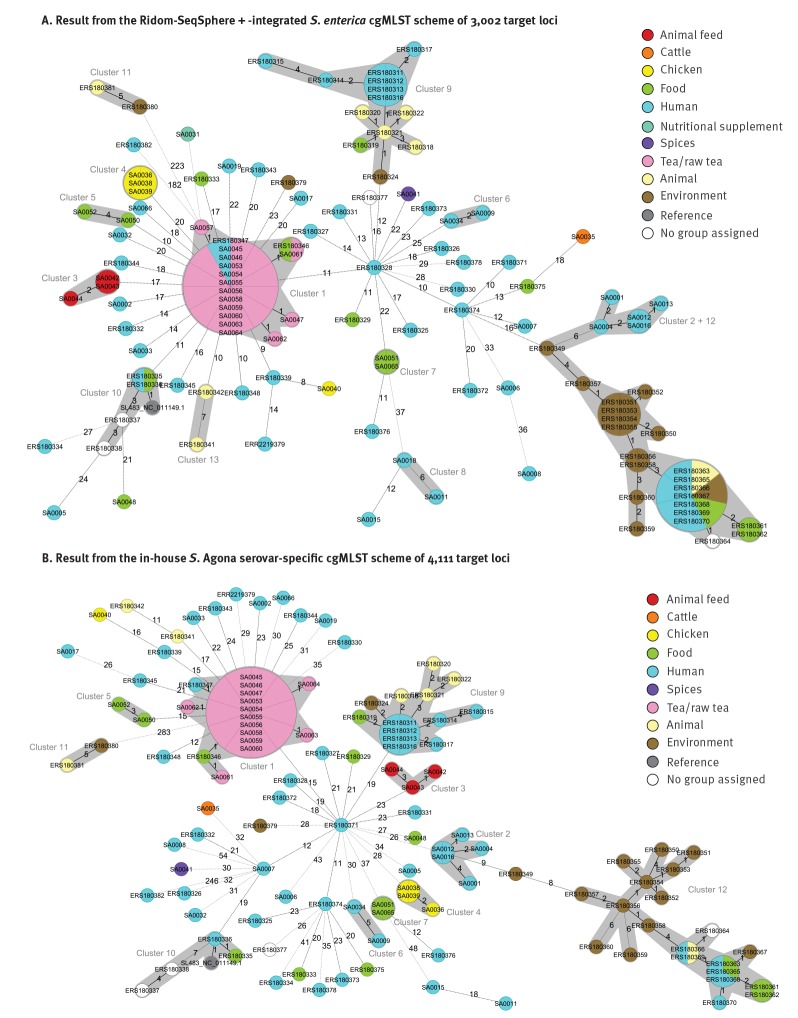
Minimum spanning trees obtained with different typing schemes (Panels A and B) of the core genome multilocus sequence type allelic profiles of 51 Bavarian *Salmonella* Agona isolates, a representative isolate of an infant milk outbreak in France, the reference strain SL-483 and 70 isolates from various European outbreaks (n = 123 isolates)^a^

To evaluate the needed resolution of the cgMLST analysis regarding analysed genomic content and consequential cluster demarcation, the species level typing with the public *S.* *enterica* cgMLST scheme, consisting of Enterobase-developed 3,002 target loci was compared with an in-house generated ad hoc serovar Agona-specific cgMLST scheme with 4,111 target loci (Figure 3). Typing with both schemes was performed using the SeqSphere + software algorithms. As no empiric threshold was defined prior the analysis for the serovar-specific scheme it was used with the same thresholds as the species-specific scheme. For the comparison of the data analysis approaches, 70 published NGS raw datasets of isolates described by Zhou et al. [21] were added in both approaches to widen the view on cluster definition and concordance by genomic and epidemiological data on a supra-regional level. This dataset covered *S.* Agona isolates from 1952 to 2010 covering several European outbreaks. The results of the public *S. enterica* cgMLST scheme (Figure 3A) are very similar to those of the in-house *S.* Agona cgMLST scheme (Figure 3B) and SNP-profiling (Figure 2). All Bavarian and published outbreak clusters were detected by both schemes (Figure 3A and B). Therefore, the usage of the public *S. enterica* scheme was generally evaluated as practical for *S.* Agona outbreak investigations.

cgMLST: core genome multilocus sequence typing.

^a^ Due to shortfall below the 90% good target threshold for the *S.* Agona scheme target loci, SA0018 does not figure in panel B, which shows a total of 122 isolates.

Bavarian *S. *Agona samples have the prefix SA. The representative isolate of the infant milk outbreak in France [23] is ERR2219379. The National Center for Biotechnology Information (NCBI) reference genome of strain SL-483 has GenBank accession number NC_011149.1. The 70 isolates from various European outbreaks are published in a separate study [21].

Allele distances between samples are indicated and clusters of samples with minimum spanning distances of zero to seven alleles are shaded in grey. Samples are colour coded by their isolation source, as given in the legend.

As SA0018 was excluded from the serovar-specific cgMLST due to shortfall below the 90% good target threshold for the *S.* Agona scheme target loci, cluster 8 detected in species-specific cgMLST (Figure 1, Figure 3A) was not apparent in serovar-specific cgMLST (Figure 3B).

Due to a smaller number of target loci in the public cgMLST scheme, allele distances between samples were generally lower than with the serovar-specific scheme. Two epidemiologically unrelated clusters linked by an unrelated Irish environmental isolate (ERS180349) (Figure 3B, clusters 2 and 12) could not be delimitated clearly in the *S.* *enterica* scheme with a default cluster threshold of seven alleles (Figure 3A, cluster 2 + 12). Those two clusters – one consisting of Bavarian patient isolates with travel history to African or Arabian countries described above (cluster 2; Table 1 and 2) and one described by Zhou et al. (Figure 3B, cluster 12), originating from a large multi-country 2008−09 outbreak originating from Ireland [21] – could more clearly be separated with the in-house cgMLST *S.* Agona scheme (Figure 3B, clusters 2 and 12), using the same cluster threshold. In the species-level cgMLST scheme, the software assigns CTs for all samples. These were different for most isolates of the two unrelated clusters, suggesting two different sources for these two clusters, additional to the epidemiological information.

## Discussion

Our high resolution WGS-based analysis approach by either wg SNP-profiling or species- or serovar-specific cgMLST delivered good and reliable results for typing *S.* Agona isolates and evaluating their affiliation to, or delineation from, outbreak clusters. We could thereby exclude a connection between the Bavarian feed sample isolates from 2017 and a large outbreak due to infant milk products, which occurred at the same time in France and other European countries. Furthermore, we could confirm *S.* Agona epidemiological clusters from former years as well as identify previously unrecognised clusters. As recently shown for serovar *S.* Enteritidis [38], cgMLST was a standardisable and easily applicable to *S.* Agona and could reach an analysis depth comparable to wg SNP profiling. 

Recently, cgMLST was published as a tool with reasonable resolution for investigations of *Salmonella* population structure [29]. Thereby, the extensively curated *S. enterica* based scheme was made available on a publicly accessible website [28]. The same scheme was implemented in the Ridom SeqSphere + Software too, although with own allele calling and clustering rules and was used in this study to compare the resolution of species- and serovar-specific typing with our in-house developed serovar-specific scheme. It could be shown that even on species level all outbreaks were clearly identified by both schemes. Only two independent outbreak clusters could not be delimitated clearly anymore by the publicly available scheme, due to the lower number of species-specific target loci and the used cluster threshold.

Of course, more focused typing schemes or approaches like serovar-level cgMLST or wg SNP genotyping deliver more detailed typing results catching at least the portion of the serovar-specific diversity manifested in the core- or reference-genome. This is particularly true for monomorphic organisms such as *S.* Agona, for which mobile elements that are mostly not covered by core genome or single reference-based approaches generate a lot of diversity [21], or for situations when single samples are to be inferred as part of specific clusters or not, especially when epidemiological information is not fully discriminative. Consequently, the serovar-specific in-house scheme developed for this investigation, worked very well for the analysed sample set. However, due to the lack of target curation and its creation from only one reference and three query genomes, it will need further optimisation and testing with diverse sample sets for suitability on a broader scale. This can be assumed from the fact that four isolates had to be excluded from typing due to shortfall below the 90% good target threshold using the in-house scheme, but only one isolate using the publicly available scheme (Table 1). The use of a publicly available scheme whose target loci underwent extensive curation, like the Enterobase *S.* *enterica* scheme [29], can render suitable and reliable results even in cases where serotyping has not yet been performed or in the event of the lack of a specific reference.

Generally, cluster definition is based on a single-linkage allelic difference threshold. The software implements a proposed default threshold of seven alleles for the *S.* *enterica* scheme. This was adopted for the *S.* Agona scheme for comparison and as no empirically tested threshold was established for this scheme before. However, as shown with the high-resolution in-house *S.* Agona cgMLST scheme, clusters with a clear epidemiological link did not show more than four alleles and six SNPs difference. Therefore, a cluster with a distance above four, but below the threshold of seven alleles (e.g. Figure 3B, clusters 6 and 11), may contain falsely grouped samples. Thus, the cluster thresholds in both schemes could be decreased down from seven based on the analysed sample set. However, theoretically a cluster threshold should be lower in a scheme with less loci like the *S. enterica* scheme (3,002 loci) than in a scheme with more loci like the *S.* Agonas scheme (4,111 loci). A decreased cluster threshold would be in accordance with the special genetic characteristics of *S.* Agona, which emerged more recently and is more monomorphic than most other serovars [21]. However, while facilitating cluster delimitation in *S.* Agona, a decreased threshold would, especially in the *S. enterica* scheme, very likely impair clustering in the case of more heterogeneous serovars or when linked isolates have evolved over a longer time period.

Cluster types assigned by the publicly available *S.* *enterica* scheme are intended to roughly classify genetically similar samples in an easy way. This is very helpful and an important step towards standardisation, also in terms of inter-laboratory communication and for alerts concerning the detection of a known CT. In the case of the two epidemiologically unrelated clusters 2 and 12 (Figure 3B), which were not clearly delimited by the public *S. enterica* scheme (Figure 3A, Cluster 2 + 12), the software-assigned CT values for each isolate were mainly distinct for the two clusters (cluster 2: CT-1195, cluster 12: CT-25). Hence, using the CT as a simplification measure to distinguish between clusters from different sources helped, although the clusters were difficult to resolve at least with a MST and the applied threshold for single-linkage clustering. However, due to the complexity of NGS data the logical principle on which such a simplification, like using the CT value, is based, always has to be considered for its correct use, as this can also result in false inclusion into or exclusion from a cluster. Indeed, the CTs are assigned depending on an isolate’s proximity in terms of allelic distance to a specific CT founder allele profile or by incremental expansion of the nomenclature when a new isolate exhibits a not yet known and sufficiently different allele combination [30,31]. The incremental nomenclature expansion together with the fact that only the distance to the nearest CT founder is reflected in the CT assignment of new isolates can lead to the assignment of the same CT to isolates with allelic distances above the threshold or of different CTs to isolates below a cluster threshold. This may be for example the case for isolate ERS180350, correctly grouped within the Irish outbreak cluster of 2008 with common CT-25 (Figure 3B, cluster 12), but being tagged with a deviant CT (CT-1224). Furthermore, microbial evolution can be different in certain epidemiological niches which cannot fully be covered by such simplification.

Due to these limitations, outbreak investigations should generally avoid relying solely on simplifications like CT values, but also include visual inspection of cluster formation in trees to avoid overlooking of connected samples. Classifications by clusters or simplified measures such as CTs should always be interpreted with caution, especially when allelic or SNP distances near an empirically tested cluster threshold occur. Moreover, particularly, but not only, for organisms with specific genetic characteristics such as *S.* Agona, empirical as well as epidemiological data should always be taken into account in addition to the molecular data [39]. 

Our analysis also shows that if a common reference or close genetic relationship for a specific set of isolates is known, high resolution approaches facilitate analysis and enable clear grouping of individual isolates in dispute, whereas the versatility of genetically broader approaches enables more standardised results for more heterogeneous sets of isolates. To reduce interpretation complexity and further extend fast and easy usability of NGS approaches for public health analyses, further standardisation and harmonisation between laboratories are nevertheless required. Many researchers and authorities have already realised this, but the implementation and ongoing optimisation will be a huge effort in the forthcoming years.

Concluding, consistent with results obtained for other serovars and species, wg SNP profiling as well as serovar- or species-specific cgMLST can be used for reasonable, reproducible and reliable high resolution classification of *S.* Agona WGS data to detect outbreak clusters. We showed this with a representative dataset from regional and international sources covering human, food, feed, veterinary and environmental isolates and thereby various types of focus areas of public health authorities. With this approach, relationships between past or international cases could also be inferred using representative public data. We also highlighted the importance and supportive power of epidemiological sample data and an integrated view on both molecular and epidemiological data. Importantly, NGS results still need careful evaluation, as their interpretation approaches often have to be a trade-off between highest resolution and versatility. Standardisation and harmonisation on an international level will further improve using the surplus of information coming from NGS in molecular surveillance.
